# Bio-Psychological Predictors of Acute and Protracted Fatigue After Burns: A Longitudinal Study

**DOI:** 10.3389/fpsyg.2021.794364

**Published:** 2022-01-24

**Authors:** Elise Boersma-van Dam, Iris M. Engelhard, Rens van de Schoot, Nancy E. E. Van Loey

**Affiliations:** ^1^Association of Dutch Burn Centres, Beverwijk, Netherlands; ^2^Department of Clinical Psychology, Faculty of Social and Behavioral Sciences, Utrecht University, Utrecht, Netherlands; ^3^Department of Methodology and Statistics, Faculty of Social and Behavioral Sciences, Utrecht University, Utrecht, Netherlands; ^4^Optentia Research Program, Faculty of Humanities, North-West University, Vanderbijlpark, South Africa; ^5^Maasstad Hospital and Association of Dutch Burn Centres, Rotterdam, Netherlands

**Keywords:** burns, fatigue, PTSD symptoms, pain, bio-psychological approach

## Abstract

**Objective:**

Fatigue after burns is often attributed to the hyperinflammatory and hypermetabolic response, while it may be best understood from a bio-psychological perspective, also involving the neuro-endocrine system. This longitudinal multi-center study examined the course of fatigue up to 18 months postburn. The contribution of bio-psychological factors, including burn severity, pain, and acute PTSD symptoms, to the course and persistence of fatigue was studied in a multifactorial model.

**Methods:**

Participants were 247 adult burn survivors. Fatigue symptoms were assessed with the Multidimensional Fatigue Inventory during the acute phase and subsequently at 3, 6, 12, and 18 months postburn, and were compared to population norms. Age, gender, burn severity, acute PTSD symptoms and pain were assessed as potential predictors of fatigue over time in a latent growth model.

**Results:**

At 18 months postburn, 46% of the burn survivors reported fatigue, including 18% with severe fatigue. In the acute phase, higher levels of fatigue were related to multiple surgeries, presence of pain, and higher levels of acute PTSD symptoms. Fatigue gradually decreased over time with minor individual differences in rate of decrease. At 18 months, pain and acute PTSD symptoms remained significant predictors of fatigue levels.

**Conclusions:**

Protracted fatigue after burns was found in almost one out of five burn survivors and was associated with both pain and acute PTSD symptoms. Early detection of PTSD symptoms and early psychological interventions aimed at reducing PTSD symptoms and pain may be warranted to reduce later fatigue symptoms.

## Background

Burn survivors often report fatigue symptoms in the aftermath of burn injuries (Dahl et al., 2012; Holavanahalli et al., 2016; Kool et al., 2017). This feeling of persistent tiredness, weakness or exhaustion can manifest mentally and physically, is not relieved by rest, and may interfere with general activities, mood, and work-related ability (Dittner et al., 2004; Gabbe et al., 2016). Fatigue is prevalent upon hospital discharge with rates between 66 and 75% (Esfahlan et al., 2010; Simko et al., 2018), including 37% with moderate to severe fatigue (Gabbe et al., 2016). Fatigue levels tend to normalize after the 1st year postburn (Edwards et al., 2007; Corry et al., 2010; Gabbe et al., 2016; Simko et al., 2018), although levels may not return to retrospective pre-burn levels (Simko et al., 2018). Generally, higher levels of fatigue were found in women compared to men and in older compared to younger burn survivors (Edwards et al., 2007; Edgar et al., 2013; Toh et al., 2015; Gabbe et al., 2016; Simko et al., 2018). However, particularly long-term fatigue after burns is still poorly understood.

Fatigue may involve multiple interacting physiological and psychosocial factors (Geenen and Dures, 2019). Its exact pathophysiology is unknown, but there is consensus that the immune system and neurological system play a key role. Pro-inflammatory cytokines disturb the neuronal environment and signal the brain to set illness-related behavioral priorities, such as fatigue, that contribute to survival and repair (Dantzer et al., 2014). Burns typically trigger a local and systemic inflammatory response, characterized by an excessive production of pro-inflammatory cytokines (Jeschke et al., 2011; Mulder et al., 2020), which intensifies with increasing burn size (Barber et al., 2008). The inflammatory response gradually decreases with time, although prolonged elevated levels have been reported (Mulder et al., 2020). In the post-acute phase, the pathophysiological stress response (Herndon and Tompkins, 2004; Jeschke et al., 2011; Porter et al., 2016) may explain the reported relation between burn severity and fatigue (Edgar et al., 2013; Gabbe et al., 2016; Simko et al., 2018), but it may fall short in explaining chronic fatigue.

In the critical illness literature, it is proposed that the endocrine system may also be involved in fatigue through dysregulation of several endocrine axes (Weekers and Van den Berghe, 2004; Stanculescu et al., 2021). The hypothalamic-pituitary adrenal axis (HPA-axis) may be of particular relevance. This fundamental physical stress response system regulates hormonal levels such as corticotropin-releasing factor (CRF) and corticosteroid levels including cortisol, in response to physical and mental challenges (Gupta et al., 2007). Over time, initially high levels of CRF gradually normalize through a feedback loop, in which elevated cortisol levels trigger HPA-axis suppression. However, prolonged suppression may occur in the aftermath of critical illness and is also observed in persons with chronic fatigue. Hence, prolonged HPA-axis suppression is presumably related to protracted fatigue (Stanculescu et al., 2021).

Besides the extensive wounds that challenge the stress system, triggers of the HPA-axis may include severe pain related to daily repetitive wound care procedures and the potentially traumatic nature of the burn event. A review showed that about 2 to 30% of burn survivors develop acute stress disorder and up to 40% may develop post-traumatic stress disorder (PTSD; American Psychiatric Association, 2013) after 3 to 6 months (Giannoni-Pastor et al., 2016). Studies have shown that both pain and PTSD symptoms are related to higher levels of fatigue after burns (Corry et al., 2010; Esfahlan et al., 2010), and both decrease with time, but are entangled (Ravn et al., 2018; Van Loey et al., 2018b). In the acute phase, PTSD symptoms and pain have been related to biological markers of stress, such as cortisol (Brown et al., 2014), and to the neuropeptide oxytocin, that is associated with HPA-axis regulation (Van Loey et al., 2018a; Yoon and Kim, 2019). Also, PTSD has been associated with immune activation, in which pro-inflammatory cytokines can act as mediators of the stress response (Yehuda et al., 2015). So far, only one longitudinal burn study has investigated pain and PTSD symptoms in relation to fatigue after burns and found a temporal effect of PTSD symptoms, but not of pain (Corry et al., 2010). In sum, pain and PTSD symptoms may exert an influence on the neuro-endocrine and immune systems involved in (chronic) fatigue.

The aim of the current longitudinal multi-center study was to test the predictive value of burn severity, pain, and acute PTSD symptoms for acute and chronic (at 18 months) fatigue symptoms, and its course over time. Based on the literature, it was expected that older age, female gender, burn severity, and higher levels of pain and acute PTSD symptoms would be related to higher initial levels of fatigue, and that particularly pain and acute PTSD symptoms would be related to protracted fatigue levels.

## Methods

### Inclusion

The data from this study were part of a larger longitudinal project in three Dutch and three Belgian burn centers. Previous work focused on quality of life in burn survivors (Boersma-van Dam et al., 2021). Patients were recruited between October 2013 and October 2015 and were followed for 18 months. Inclusion criteria were: hospital stay of >24 h following the burn event, age of 18 years or older, and proficiency in Dutch. Exclusion criteria were: psychiatric problems that may interfere with the comprehension or completion of questionnaires (e.g., psychosis, cognitive problems).

### Procedure

Patients were invited to participate in the study by a local researcher during their stay in the burn center. After they received oral and written information about the study and agreed to participate, they provided written informed consent. Patients completed the first assessment in the acute phase and follow-ups at 3, 6, 12, and 18 months postburn by postal mail. The study was approved by ethics boards in the Netherlands and Belgium (NL44682.094.13 and B670201420373).

### Sample and Missing Data

Of the 266 burn survivors enrolled in the larger study, 247 were included in the analyses. They were admitted to the burn center in Groningen (*n* = 42), Beverwijk (*n* = 49), Rotterdam (*n* = 97), Antwerp (*n* = 16), Ghent (*n* = 17), or Brussels (*n* = 26). These 247 burn survivors completed at least one General Fatigue measure *and* completed all predictor measures at the first assessment. Thirteen burn survivors did not complete any of the General Fatigue measures and six missed information on at least one predictor. The 247 burn survivors did not differ significantly (*p*s > 0.05) from the excluded 19 burn survivors in terms of age, gender, TBSA, and number of surgeries.

The number of burn survivors that completed General Fatigue measurements in the acute phase (denoted as T1), and at 3 (T2), 6 (T3), 12 (T4), and 18 (T5) months postburn was 246 (99.6%), 212 (85.8%), 198 (80.2%), 165 (66.8%), and 156 (63.2%), respectively. A total of 141 (57.1%) burn survivors completed all five measurements. Burn survivors with partially missing fatigue data (*n* = 106) did not differ significantly (*p* > 0.05) from burn survivors with complete data (*n* = 141) in terms of gender, TBSA, number of surgeries, duration of mechanical ventilation, acute PTSD symptoms, and General Fatigue at T1. However, those with partially missing fatigue data were significantly younger than those with complete data (*M* = 41.4, *SD* = 15.5 vs. *M* = 46.0, *SD* = 15.2), *t*(245) = −2.3, *p* = 0.022, and reported significantly less pain (20.8% reported no pain vs. 7.8% in those with complete data), χ^2^(2) = 10.1, *p* = 0.007.

### Measures

#### Fatigue

The Multidimensional Fatigue Inventory (MFI-20; Smets et al., 1995) is a self-report questionnaire with five dimensions, i.e., General Fatigue, Physical Fatigue, Reduced Activity, Reduced Motivation and Mental Fatigue. It was assessed at all five measurement points. The 5-point Likert scale items were summed for each dimension, with higher scores indicating higher levels of fatigue. General Fatigue results were reported and analyzed, as recommended in the manual and in line with common definitions of fatigue. Results regarding the other dimensions are included in the Supplementary Material. The MFI-20 was tested and validated in several Dutch patient groups (Smets et al., 1995). Reliability of General Fatigue in the current study was good, with Cronbach’s alpha ranging from 0.87 to 0.90 over time.

#### Pain

The pain item of the EQ-5D-3L (Rabin and de Charro, 2001) self-report scale was used to assess overall pain in the acute phase. Patients rated whether they experienced “no pain or discomfort”, “moderate pain or discomfort”, or “extreme pain or discomfort”. The EQ-5D has good feasibility and reasonable criterion validity in the burn population (Öster et al., 2009).

#### Post-traumatic Stress Disorder Symptoms

The Impact of Event Scale-Revised (IES-R; Weiss and Marmar, 1997; van der Ploeg et al., 2004) is a 22-item self-report questionnaire that was used to asses symptoms of intrusions, avoidance, and hyper-arousal in the acute phase (T1). Answers were given on a 5-point Likert scale and summed to obtain a total score ranging from 0 to 88. This questionnaire cannot be used to diagnose PTSD, but a score of 33 or higher may reflect a possible diagnosis of PTSD. If at least 20 of the 22 items were completed, sum scores were calculated based on the mean of the completed items. The IES-R has been validated in Dutch trauma populations and showed good psychometric properties (Olde et al., 2006).

#### Demographics and Burn Characteristics

Age, gender, number of surgeries and total body surface area (TBSA) burned were recorded from the patient’s medical file. Number of surgeries indicates the number of skin graft procedures that was required to cover the wounds and is considered an indicator of burn severity. TBSA is the estimated percentage of the body covered with partial and full thickness burns.

### Statistical Analysis

Descriptive analyses were conducted in IBM SPSS v24. Continuous predictors were correlated with General Fatigue at each time point. Categorical predictors were related to General Fatigue at each time point using ANOVA’s and (*post hoc*) *t*-tests. To correct for multiple testing in the (*post hoc*) *t*-tests, the Benjamini-Hochberg multiple testing procedure was followed (Raykov et al., 2013). That is, we calculated a corrected alpha value (*l*). The *p*-values of the (*post hoc*) *t*-test should then be smaller than *l* instead of the default alpha of 0.05. The formula for obtaining *l* is provided in the footnote of Table 2. To determine the prevalence of fatigue, scores were compared to the age and gender adjusted mean, 75th percentile (moderate fatigue) and 90th percentile (severe fatigue) of a national representative sample of the non-institutionalized adult population of Germany (Schwarz et al., 2003). *T*-tests were used to test the mean difference of the General Fatigue scores at each time point and the adjusted norm score for significance against 0.

**TABLE 1:** Number and percentage of burn survivors reporting fatigue, moderate-severe fatigue and severe general fatigue at each measurement.

	Fatigue > mean^1^		Moderate-severe fatigue > 75th percentile^1^		Severe fatigue > 90th percentile^1^	
Measurement	*n*	%	*n*	%	*n*	%
Acute phase	185	75.2	155	63.0	108	43.9
3 months	141	66.5	112	52.8	83	39.2
6 months	119	60.1	88	44.4	54	27.3
12 months	86	52.1	58	35.2	33	20.0
18 months	72	46.2	48	30.8	28	17.9

*^1^Individual scores were compared to, respectively, the mean, 75th percentile, and 90th percentile of gender and age adjusted population norms (Schwarz et al., 2003).*

**TABLE 2:** Descriptives and bivariate pearson correlation matrix of study variables and general fatigue.

	(1)	(2)	(3)	(4)	(5)	(6)	(7)
(1) Age							
(2) PTSD symptoms	−0.08						
(3) General fatigue T1	−0.05	0.39**					
(4) General fatigue T2	−0.02	0.39**	0.52**				
(5) General fatigue T3	−0.10	0.43**	0.48**	0.69**			
(6) General fatigue T4	−0.03	0.41**	0.47**	0.60**	0.64**		
(7) General fatigue T5	−0.00	0.41**	0.44**	0.58**	0.67**	0.65**	
N	247	247	246	212	198	165	156
Mean	44.0	18.0	12.0	11.0	10.1	9.2	8.9
Gender^1^							
Male (*n* = 176)	44.2	14.8^a^	11.3^a^	10.3^a^	9.6	8.6	8.2
Female (*n* = 71)	43.5	25.8^b^	13.6^b^	12.7^b^	11.2	10.6	10.4
Surgeries^1^							
0 surgeries (*n* = 118)	41.9	17.3	11.7	9.9^a^	9.3^a^	8.5	8.0
1 surgery (*n* = 87)	45.0	17.3	12.0	11.4^a,b^	10.1^a,b^	9.5	9.4
>1 surgeries (*n* = 42)	47.9	21.3	12.7	13.2^b^	12.0^b^	10.3	10.0
Pain^1^							
None (*n* = 33)	43.1	10.3^a^	9.0^a^	8.4^a^	7.7^a^	6.4^a^	6.6^a^
Moderate (*n* = 169)	45.0	16.4^a^	11.7^b^	10.8^a^	10.0^a,b^	9.0^a^	8.6^a,b^
Severe (*n* = 45)	40.9	29.5^b^	15.2^c^	13.3^b^	12.0^b^	11.6^b^	11.1^b^
SD	15.5	16.7	4.7	5.0	4.7	4.7	4.5
Median	43	12	12	11	10	9	8

** p ≤ 0.010.

*^1^Sample size at T1.*

*^abc^ Groups with non-identical superscripts differ significantly with p < l;*

l = (0.05/(31 * (1/1 + 1/2 + 1/3 + […] + 1/31))) * c where c = 1, 2, 3, […], 31 for the ranked p-values to obtain a new alpha value for each new t-test. Post hoc t-tests for surgeries and pain were only performed if the ANOVA yielded significant results.

*PTSD = Post-traumatic stress disorder.*

*T1 = Acute, T2 = 3 months, T3 = 6 months, T4 = 12 months, T5 = 18 months postburn.*

Longitudinal trajectories of General Fatigue were estimated using Latent Growth Modeling (LGM) in Mplus 8.5 (Muthén and Muthén, 1998–2017). Full Information Maximum Likelihood (FIML) was used to handle missing data in the model. By including the observed variables related to the probability of missingness in the model, FIML leads to unbiased parameter estimates (Enders, 2010; Hox, 2010). To account for the non-normality of some of the variables, Robust Maximum Likelihood (MLR) was used. Model fit of nested models was compared using adjusted chi-square difference tests (Satorra and Bentler, 2001).

Three consecutive growth models were estimated. First, a linear growth model was constructed with the slope growth factors representing the timing of the measurements since the burn event. Second, the addition of a quadratic term was evaluated, to determine the best fitting shape of the curve. Third, predictors were added to the best fitting growth model, i.e., gender, age, number of surgeries, pain, and acute PTSD symptoms were regressed on the intercept (starting point at T1) and the slope. Age and acute PTSD symptoms were grand-mean centered to aid interpretation of the intercept and slope estimates. Also, to investigate the relevance of the predictors for protracted symptoms of fatigue, we reran the final growth model, but changed the specifications such that the intercept became the endpoint at 18 months postburn.

Model fit was evaluated with the comparative fit index (CFI), the Tucker-Lewis Index (TLI), and the root mean square error of approximation (RMSEA). Models with a TLI and CFI > 0.90 and RMSEA < 0.08 indicate an acceptable fit, and models with TLI and CFI values > 0.95 and RMSEA values < 0.05 indicate a good fit to the data (Kline, 2011).

## Results

### Descriptive Analyses

The sample of 247 burn survivors included predominantly men (*n* = 176, 71.3%), and had a mean age of 44.0 years (*SD* = 15.5, range: 18–82). Mean TBSA was 9.2% (*SD* = 11.1, range: 1 – 75). Median number of surgeries was 1 (range 0 – 14). For further analyses, this variable was recoded into “no surgeries” (*n* = 118; 47.8%), “one surgery” (*n* = 87; 35.2%) or “more than one surgery” (*n* = 42; 17.0%). Twenty-nine (11.7%) burn survivors had received mechanical ventilation with a mean duration of 9 days (*SD* = 10.5, *Mdn* = 4, range 1–39). In the acute phase, 33 (13.4%) burn survivors reported no pain, 169 (68.4%) moderate pain, and 45 (18.2%) severe pain or discomfort. Forty-four (17.8%) burn survivors showed acute PTSD symptoms within the clinical range.

**FIGURE 1:**
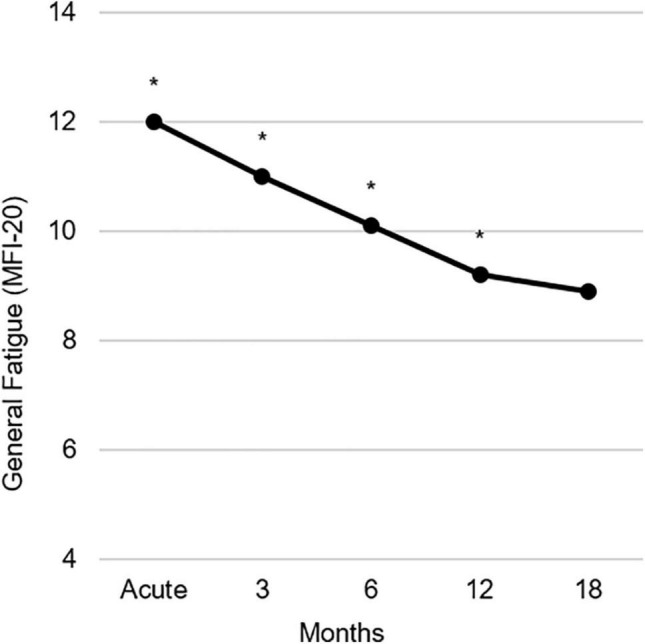
Observed course of the mean General Fatigue measured with the multidimensional fatigue inventory (MFI-20) during 18 months postburn. Asterisks indicate significant differences from population norms (Schwarz et al., 2003), with *p*s ≤ 0.01.

Table 1 shows the prevalence of General Fatigue at three severity levels compared to population norms (Schwarz et al., 2003). In the acute phase, 75.2% of the burn survivors reported fatigue (> mean of the general population), including 43.9% reporting severe fatigue (> 90th percentile of the general population). Over time, these percentages decreased to, respectively, 46.2 and 17.9% at 18 months postburn. At 18 months, moderate to severe and severe fatigue prevalence rates were about 6% and 8% higher than in the general population (25 and 10%, respectively). A comparison between all fatigue dimensions and population norms can be found in the Supplementary Material (Supplementary Figure 1 and Supplementary Tables 1, 2). Figure 1 shows the mean General Fatigue score over time. Fatigue was highest during the acute phase and showed a significant decrease within 18 months (*p* < 0.001). Nevertheless, General Fatigue scores were significantly higher than general population norms from the acute phase up to 12 months postburn.

Table 2 presents bivariate associations between the predictors and General Fatigue scores over time. Pearson correlations between age and General Fatigue were not statistically significant, whereas correlations between acute PTSD symptoms and General Fatigue all were. Women showed significantly higher levels of General Fatigue in the acute phase and at 3 months (Cohen’s *d*s = 0.5) and a higher level of acute PTSD symptoms (*d* = 0.7) compared to men. Those with severe pain showed significantly higher levels of fatigue over time than those with no pain (*d* ranged from 1.0 – 1.6) and reported significantly more acute PTSD symptoms than those with no pain (*d* = 1.1) and moderate pain (*d* = 0.7). Also, women reported relatively more severe pain than men, χ^2^ (2) = 6.9, *p* = 0.031. At 3 and 6 months postburn, General Fatigue was significantly higher in those with multiple surgeries than those with no surgeries (*d* = 0.7 and *d* = 0.6, respectively). Finally, having received mechanical ventilation was significantly related to higher levels of fatigue in the acute phase, *t*(244) = −2.2, *p* = 0.030, and at 6 months postburn, *t*(196) = −2.4, *p* = 0.017, to higher levels of acute PTSD symptoms, *t*(245) = −2.0, *p* = 0.044, and to multiple surgeries, χ^2^(2) = 22.8, *p* < 0.001.

### Latent Growth Modeling

A linear growth model approximated the thresholds for acceptable fit to the General Fatigue data, χ^2^ (10) = 29.41, *p* = 0.001; RMSEA = 0.089; CFI/TLI = 0.933. The quadratic model that was tested produced an impossible high negative correlation (*r* < −1.0) between the linear and quadratic slope, indicating that this model was not reliable. Therefore, predictors were added to the linear growth model. The results are shown in Table 3. Addition of the predictor variables resulted in a model with an acceptable model fit, χ^2^(31) = 56.45, *p* = 0.004; RMSEA = 0.058; CFI = 0.942; TLI = 0.916. This model accounted for 38% of the variance in fatigue in the acute phase, and 3% of the variance around the decline in fatigue over time. With regard to the intercept, burn survivors with multiple surgeries, higher levels of pain or acute PTSD symptoms, reported higher levels of fatigue, compared to burn survivors who did not need surgeries, or who reported lower levels of pain or acute PTSD symptoms, respectively. No significant associations were found between the predictors and the slope. When the variance around the slope in the model without predictors was constrained to zero, this resulted in a non-significant difference in model fit compared to the original model, Δχ^2^_SB_(2) = 1.26, *p* = 0.12, indicating little variability in change of fatigue scores over time between burn survivors.

**TABLE 3:** Linear growth over time: predictors of general fatigue (*N* = 247).

	Intercept acute phase			Slope			Endpoint 18 months		
	Estimate	SE	*p*	Estimate	SE	*p*	Estimate	SE	*p*
Correlation with slope	–0.19	0.17	0.27				0.56	0.12	<0.001
Intercept	13.62	0.79	<0.001	–1.39	0.54	0.01	6.39	0.80	<0.001
Gender^1^	0.78	0.57	0.17	0.08	0.43	0.85	0.92	0.70	0.19
Age	–0.01	0.02	0.61	0.01	0.01	0.28	0.02	0.02	0.40
Surgeries									
1 vs. 0	0.47	0.53	0.37	–0.09	0.40	0.83	0.31	0.66	0.64
>1 vs. 0	1.46	0.72	0.043	–0.36	0.58	0.54	0.82	0.94	0.39
>1 vs. 1	0.99	0.75	0.19	–0.27	0.58	0.64	0.51	1.00	0.61
Pain									
No vs. Moderate	1.87	0.72	0.009	–0.27	0.55	0.63	1.39	0.84	0.10
No vs. Severe	3.26	0.89	<0.001	–0.36	0.77	0.64	2.62	1.22	0.032
Moderate vs. Severe	1.39	0.62	0.024	–0.09	0.57	0.87	1.22	0.94	0.20
Acute PTSD symptoms	0.09	0.02	<0.001	0.01	0.01	0.61	0.10	0.02	<0.001

*PTSD = Post-traumatic stress disorder.*

*^1^Male is the reference category.*

To investigate the relevance of the predictors for long-term symptoms of fatigue, we reran the model such that the endpoint at 18 months postburn was predicted instead of the intercept (acute phase). This adjusted model accounted for 31% of the variance in fatigue at 18 months postburn. Table 3 shows that burn survivors with extreme levels of acute pain and those with higher acute PTSD symptoms, reported higher levels of fatigue at 18 months postburn compared to those who reported no acute pain or lower acute PTSD symptoms, respectively. See also Supplementary Table 3 for the linear growth modeling results of Mental Fatigue.

## Discussion

This longitudinal study examined the prevalence, course and possible predictors of fatigue in burn survivors. The estimated prevalence of (severe) fatigue was high in the acute phase, and decreased considerably over time. Initial higher levels of fatigue were predicted by multiple surgeries, extreme pain and higher levels of acute PTSD symptoms. Higher levels of fatigue at 18 months were predicted by extreme pain and higher levels of acute PTSD symptoms. None of the predictors were associated with the rate of decline in fatigue over time.

The estimated prevalence of fatigue of around 75% (44% severe) in the acute phase, and 46% (18% severe) at 18 months are consistent with previous studies in burn survivors (Gabbe et al., 2016; Simko et al., 2018). At 18 months postburn, the average fatigue level was similar to that of the general population and 6 months fatigue prevalence rates were comparable to chronic critally ill patients (Wintermann et al., 2018). Still, almost one in five burn survivors continued to report severe fatigue at 18 months postburn. Previous research showed that, as a group, burn survivors did not return to retrospectively assessed pre-burn levels of fatigue and quality of life (Simko et al., 2018; Boersma-van Dam et al., 2021) and that population norms may be unrepresentative of pre-trauma health (Wilson et al., 2012). Together, these findings emphasize the need to look beyond mean population levels to accurately describe health and recovery after burns over time and call for research aimed to establish adequate cutt-offs for early screening of moderate and severe fatigue in burn survivors.

As hypothesized, fatigue was related to both biological and psychological factors. Burn severity was a robust predictor of fatigue in the acute phase, in line with previous longitudinal studies (Edgar et al., 2013; Toh et al., 2015; Gabbe et al., 2016; Simko et al., 2018), but not of protracted fatigue. This finding subscribes previous research showing a relation between TBSA and fatigue up to 12 months postburn, but not beyond (Simko et al., 2018). The initial effect of burn severity on fatigue may be explained by the hyper-metabolic and hyper-inflammatory responses in severe burns that attenuate after the sub-acute phase, but also by other factors associated with burn severity, i.e., burn survivors with multiple surgeries were more likely to have received mechanical ventilation, which in turn was related to higher acute PTSD symptom levels. Both mechanical ventilation and PTSD symptoms are characteristics of post-intensive care syndrom (PICS) that also encompasses fatigue (Lee et al., 2020; Stanculescu et al., 2021). More (biological) research to disentangle the influence of these factors may be indicated to move the field forward.

Overall pain and acute PTSD symptoms were significantly related to fatigue over time. The relationship between pain and fatigue is well established in patient populations with a chronic disease, such as rheumatoid arthritis, and cancer (Madsen et al., 2016; Ma et al., 2020), but hardly studied in the burn population. Pain resulting from burns may continue along with scar formation (Bijlard et al., 2017), and hence may remain associated with fatigue over time. Although pain and acute PTSD symptoms were related, both factors were unique predictors of fatigue in the multivariate model, indicating that each has a unique relationship with fatigue. In contrast, Corry et al. (2010) did not find a unique effect of pain after controlling for PTSD symptoms, pre-burn health and functioning, indicating that the effect of PTSD symptoms may be more robust than that of pain. A burgeoning body of evidence points to the connection between pain and PTSD (Fishbain et al., 2017; Ravn et al., 2018) and their association with fatigue over time (Astill Wright et al., 2020). These relations may point to the role of dysregulated endocrine axes, as proposed by Stanculescu et al. (2021), and prolonged activation of the immune system, as both pain and PTSD symptoms become chronic in a subgroup of patients (Yehuda et al., 2015). Future research could investigate whether a lasting relation between pain and PTSD symptoms with fatigue can be explained by prolonged suppression of the HPA-system and crosstalk with other bio-active components.

Finally, our results partly supported the general finding that women reported higher levels of fatigue than men (van’t Leven et al., 2010; Engberg et al., 2017), although the bivariate relation between fatigue and female gender disappeared in the longitudinal prediction model that included also pain and acute PTSD symptoms. Indeed, prevalence rates of PTSD and pain levels are generally higher in women (Brewin et al., 2000; Bartley and Fillingim, 2013), therefore future research may investigate whether pain and PTSD symptoms partly explain gender differences in fatigue.

Of notice, the decrease in fatigue over time was similar for all burn survivors and none of the predictors interfered with the amount of decrease in fatigue, indicating there was no difference in the course of fatigue for burn survivors with surgeries, more pain or higher acute PTSD symptom levels compared to their counterparts. This finding suggests that early interventions to reduce the initial psychological impact of burns may have the potency to reduce lasting effects on fatigue levels.

Strengths of the present study include the bio-psychological perspective, the longitudinal design, and the investigation of short- and long-term effects. However, some limitations should be noted. First, the sample size was relatively small and the drop-out rate was considerable, which may have affected statistical power, especially to find significant effects on the slope. Second, no information was available on pre-burn fatigue, nor on possible treatments for PTSD symptoms or fatigue that took place during the study period. Third, fatigue may be influenced by many other factors, such as sleep quality, depression, long-term impairment, physical fitness, and weight, which were not included in this study. Fourth, PTSD symptoms were not studied beyond the acute phase, although symptoms may be related to fatigue over time. Finally, acute PTSD symptoms were assessed with a valid and reliable self-report questionnaire, but questionnaires are known to overestimate PTSD rates compared to a clinical interview (Engelhard et al., 2007).

This study yields some potential clinical implications. First, it calls for monitoring fatigue, especially in more severely burned patients, those with high pain scores and acute PTSD symptoms. Psychological interventions, such as cognitive behavioral therapy and graded exercise therapy are generally effective in decreasing chronic fatigue (Yancey and Thomas, 2012). The mutual relations between pain, PTSD symptoms and fatigue emphasize the need to address all these factors in an early stage to improve burn survivors’ health. In the acute phase, burn survivors may benefit from additional non-pharmacological pain treatment (Abdi and Zhou, 2002; de Jong et al., 2007) and early treatment focused at acute PTSD symptoms (Birk et al., 2019; Fauerbach et al., 2020). More research is needed on the effects of early interventions on fatigue, functioning and mental health after burns.

In conclusion, fatigue rates after burns are considerable and decrease slowly over time. Protracted fatigue may occur, especially in those with higher levels of pain and PTSD symptoms. More attention for fatigue after burns is needed, and psychological interventions may be used to ameliorate fatigue.

## Data Availability Statement

The dataset presented in this article are not readily available because burns are treated in a limited number of dedicated burn centers in the Netherlands and Belgium, which may compromise the privacy of research participants. Requests to access the dataset should be directed to NV, nvanloey@burns.nl.

## Ethics Statement

The study involving human participants was reviewed and approved by METC Noord-Holland, the Netherlands (NL44682.094.13) and Commissie voor Medische Ethiek Universiteit Gent, Belgium (B670201420373). The patients/participants provided their written informed consent to participate in this study.

## Author Contributions

EB-VD, IE, RS, and NV designed the study. EB-VD, RS, and NV analyzed the data. EB-VD drafted the initial article. RS, IE, and NV provided feedback on the manuscript. All authors reviewed and revised the article and read and approved the final manuscript.

## Conflict of Interest

The authors declare that the research was conducted in the absence of any commercial or financial relationships that could be construed as a potential conflict of interest.

## Publisher’s Note

All claims expressed in this article are solely those of the authors and do not necessarily represent those of their affiliated organizations, or those of the publisher, the editors and the reviewers. Any product that may be evaluated in this article, or claim that may be made by its manufacturer, is not guaranteed or endorsed by the publisher.
