# Supplementary material for: Bio-Psychological Predictors of Acute and Protracted Fatigue After Burns: A Longitudinal Study
Source: Front Psychol. 2022 Jan 24;12:794364. doi: 10.3389/fpsyg.2021.794364 (PMC8818679; doi:10.3389/fpsyg.2021.794364)
Supplement: Supplementary file 1 [file Data_Sheet_1.docx]

**SUPPLEMENTARY MATERIAL**

**A comparison of all five MFI-20 dimensions**

| **SUPPLEMENTARY FIGURE 1 \|** Observed course of mean fatigue levels for each dimension of the Multidimensional Fatigue Inventory (MFI-20) during 18 months post-burn. |
| --- |

Supplementary Figure 1 shows the means for the five fatigue dimensions of the MFI-20 (Smets et al., 1995) over time. For each dimension, fatigue levels were highest during the acute phase and showed a significant decrease within the study period (*p* < .001 for each dimension). Supplementary Table 1 shows the means for the five fatigue dimensions of the MFI-20 over time. Comparisons between dimensions at each measurement occasion showed that General Fatigue was significantly higher than the other dimensions over time, except for Reduced Activity in the acute phase. Compared to the general population norms (Schwarz et al., 2003), the mean scores for General, Physical and Mental Fatigue were significantly higher from the acute phase up to 6 months post-burn; for Reduced Motivation and Reduced Activities this was true up to 3 months post-burn (see Supplementary Table 1). As of 12 months post-burn, none of the mean differences between the sample and the general population norm were statistically significant for any of the dimensions.

| **SUPPLEMENTARY TABLE 1 \|** Descriptive statistics and comparisons of the MFI-20 subscale scores at each time point. | | | | | | |
| --- | --- | --- | --- | --- | --- | --- |
|  | General Fatigue | Physical Fatigue | Reduced Activity | Reduced Motivation | Mental Fatigue | Sum Score |
| Acute Phase |  |  |  |  |  |  |
| N | 246 | 245 | 240 | 242 | 243 | 232 |
| Mean | 12.0^a*^ | 12.0^a*^ | 13.2^*^ | 9.2^b*^ | 9.4^b*^ | 55.5 |
| Median | 12.0 | 12.0 | 13.5 | 8.0 | 9.0 | 55.0 |
| SD | 4.7 | 4.9 | 4.2 | 4.2 | 4.5 | 17.9 |
| Skewness | 0.04 | -0.05 | -0.4 | 0.6 | 0.5 | 0.1 |
| 3 Months |  |  |  |  |  |  |
| N | 212 | 213 | 212 | 214 | 211 | 206 |
| Mean | 11.0^*^ | 10.1^*^ | 9.5^a*^ | 8.4^b*^ | 8.8^ab*^ | 47.8 |
| Median | 11.0 | 10.0 | 9.0 | 8.0 | 8.0 | 45.0 |
| SD | 5.0 | 4.9 | 4.5 | 4.0 | 4.6 | 20.0 |
| Skewness | 0.1 | 0.3 | 0.5 | 0.8 | 0.6 | 0.3 |
| 6 Months |  |  |  |  |  |  |
| N | 198 | 196 | 196 | 197 | 196 | 191 |
| Mean | 10.1^*^ | 9.4^a*^ | 8.6^b^ | 7.8 | 8.6^ab*^ | 44.6 |
| Median | 10.0 | 8.0 | 8.0 | 7.0 | 8.0 | 43.0 |
| SD | 4.7 | 4.6 | 4.2 | 3.8 | 4.6 | 18.6 |
| Skewness | 0.4 | 0.6 | 0.9 | 1.2 | 0.8 | 0.7 |
| 12 Months |  |  |  |  |  |  |
| N | 165 | 169 | 168 | 170 | 170 | 162 |
| Mean | 9.2 | 8.5^a^ | 8.0^a^ | 7.3^b^ | 7.8^ab^ | 41.0 |
| Median | 9.0 | 8.0 | 7.0 | 6.0 | 6.0 | 38.0 |
| SD | 4.7 | 4.3 | 4.0 | 3.6 | 4.4 | 18.0 |
| Skewness | 0.6 | 0.7 | 1.1 | 1.4 | 1.1 | 0.9 |
| 18 Months |  |  |  |  |  |  |
| N | 156 | 159 | 159 | 159 | 159 | 153 |
| Mean | 8.9 | 8.1^a^ | 7.5^bc^ | 7.2^c^ | 7.9^ab^ | 39.8 |
| Median | 8.0 | 7.0 | 7.0 | 6.0 | 6.0 | 37.0 |
| SD | 4.5 | 4.2 | 3.7 | 3.7 | 4.4 | 18.2 |
| Skewness | 0.7 | 1.0 | 1.1 | 1.3 | 0.9 | 1.0 |
| *Note.*  ^abc^ At each measurement occasion, subscale means with identical superscripts do not differ significantly, with *p* ≤  *l* ; *l* = (0.05/(50*(1/1+1/2+1/3+1/4+ …+1/50)))*c where c = 1,…,50 for the ranked p-values to obtain a new alpha value for each new test. See the Benjamini-Hochberg procedure (Raykov et al., 2013).  ^*^ Subscale mean differs significantly from the general population norm (Schwarz et al., 2003), with *p* <  *q* ; *q* = (0.05/(25*(1/1+1/2+1/3+1/4+ … +1/25)))*c where c = 1,…,25 for the ranked p-values to obtain a new alpha value for each new test. | | | | | | |

Supplementary Table 2 shows the prevalence of fatigue and severity for each dimension compared to population norms.

| **SUPPLEMENTARY TABLE 2 \|** Number and percentage of burn survivors reporting fatigue, moderate-severe fatigue and severe fatigue at each measurement occasion. | | | | | | | |
| --- | --- | --- | --- | --- | --- | --- | --- |
|  | Fatigue  (> Mean) | | Moderate-Severe Fatigue  (> 75^th^ percentile) | | Severe Fatigue  (> 90^th^ percentile) | |  |
|  | n | % | n | % | n | % |  |
| General Fatigue |  |  |  |  |  |  |  |
| Acute | 185 | 75.2 | 155 | 63.0 | 108 | 43.9 |  |
| 3 months | 141 | 66.5 | 112 | 52.8 | 83 | 39.2 |  |
| 6 months | 119 | 60.1 | 88 | 44.4 | 54 | 27.3 |  |
| 12 months | 86 | 52.1 | 58 | 35.2 | 33 | 20.0 |  |
| 18 months | 72 | 46.2 | 48 | 30.8 | 28 | 17.9 |  |
| Physical Fatigue |  |  |  |  |  |  |  |
| Acute | 185 | 75.5 | 156 | 63.7 | 108 | 44.1 |  |
| 3 months | 133 | 62.4 | 97 | 45.5 | 63 | 29.6 |  |
| 6 months | 111 | 56.6 | 83 | 42.3 | 39 | 19.9 |  |
| 12 months | 80 | 47.3 | 57 | 33.7 | 26 | 15.4 |  |
| 18 months | 67 | 42.1 | 48 | 30.2 | 23 | 14.5 |  |
| Reduced Activity |  |  |  |  |  |  |  |
| Acute | 209 | 87.1 | 185 | 77.1 | 135 | 56.3 |  |
| 3 months | 121 | 57.1 | 88 | 41.5 | 52 | 24.5 |  |
| 6 months | 88 | 44.9 | 57 | 29.1 | 32 | 16.3 |  |
| 12 months | 70 | 41.7 | 43 | 25.6 | 20 | 11.9 |  |
| 18 months | 59 | 37.1 | 32 | 20.1 | 12 | 7.5 |  |
| Reduced Motivation |  |  |  |  |  |  |  |
| Acute | 136 | 56.2 | 89 | 36.8 | 58 | 24.0 |  |
| 3 months | 106 | 49.5 | 63 | 29.4 | 47 | 22.0 |  |
| 6 months | 87 | 44.2 | 47 | 23.9 | 26 | 13.2 |  |
| 12 months | 63 | 37.1 | 26 | 15.3 | 16 | 9.4 |  |
| 18 months | 59 | 37.1 | 27 | 17.0 | 16 | 10.1 |  |
| Mental Fatigue |  |  |  |  |  |  |  |
| Acute | 147 | 60.5 | 111 | 45.7 | 72 | 29.6 |  |
| 3 months | 108 | 51.2 | 87 | 41.2 | 62 | 29.4 |  |
| 6 months | 99 | 50.5 | 72 | 36.7 | 53 | 27.0 |  |
| 12 months | 69 | 40.6 | 53 | 31.2 | 31 | 18.2 |  |
| 18 months | 71 | 44.7 | 50 | 31.4 | 31 | 19.5 |  |
| *Note.* Number and percentages of burn survivors scoring above the mean, 75^th^ and 90^th^ percentile of the gender and age adjusted population norms (Schwarz et al., 2003) | | | | | | |  |

In the acute phase, 75.2% of the burn survivors experienced General Fatigue, and 43.9% experienced severe General Fatigue compared to the general population. Over time, these percentages decreased to respectively 46.2% and 17.9% at 18 months post-burn. In the acute phase, the highest prevalence was found for Reduced Activity. At 18 months, General Fatigue and Mental Fatigue had the highest prevalence. For the dimensions General, Physical, and Mental Fatigue, 18 month moderate to severe fatigue prevalence rates were about 6% higher than in the general population (25%), and prevalence rates of severe fatigue were respectively 5%, 8% and 10% higher than in the general population (10%).

**Latent growth modeling**

| **SUPPLEMENTARY TABLE 3 \|** Linear Growth over Time: Predictors of Mental Fatigue. | | | | | | | | | | | |
| --- | --- | --- | --- | --- | --- | --- | --- | --- | --- | --- | --- |
|  | Intercept Acute Phase | | |  | Slope | | |  | Endpoint 18 months | | |
|  | Estimate | SE | *p* |  | Estimate | SE | *p* |  | Estimate | SE | *p* |
| Correlation with Slope | -0.18 | 0.12 | .13 |  |  |  |  |  | -0.77 | 0.05 | <.001 |
| Intercept | 7.93 | 0.64 | <.001 |  | -0.90 | 0.49 | .068 |  | 6.31 | 0.75 | <.001 |
| Gender | 0.31 | 0.53 | .56 |  | -0.32 | 0.42 | .44 |  | -0.27 | 0.71 | .70 |
| Age | -0.02 | 0.01 | .093 |  | 0.02 | 0.01 | .20 |  | 0.00 | 0.02 | .89 |
| Surgeries |  |  |  |  |  |  |  |  |  |  |  |
| 1 vs 0 | 0.53 | 0.48 | .27 |  | 0.32 | 0.39 | .42 |  | 1.10 | 0.66 | .098 |
| >1 vs 0 | 0.85 | 0.63 | .17 |  | -0.14 | 0.52 | .79 |  | 0.60 | 0.87 | .49 |
| >1 vs 1 | 0.32 | 0.67 | .63 |  | -0.46 | 0.54 | .40 |  | -0.50 | 0.90 | .58 |
| Pain |  |  |  |  |  |  |  |  |  |  |  |
| Moderate vs No | 0.78 | 0.66 | .24 |  | -0.05 | 0.47 | .91 |  | 0.69 | 0.74 | .35 |
| Severe vs No | 0.52 | 0.86 | .54 |  | 1.58 | 0.79 | .046 |  | 3.37 | 1.27 | .008 |
| Severe vs Moderate | 0.26 | 0.62 | .68 |  | 1.63 | 0.67 | .014 |  | 2.68 | 1.10 | .014 |
| Acute PTSD symptoms | 0.12 | 0.02 | <.001 |  | -0.02 | 0.01 | .15 |  | 0.09 | 0.02 | <.001 |
| PTSD = Post-traumatic Stress Disorder | | | | | | | | | | | |

The linear growth model that was reported in this article was also fitted to the other MFI-20 dimensions, but this resulted in bad model fit (and problems with negative residuals) for Physical Fatigue, Reduced Activity and Reduced Motivation. For Mental Fatigue, a linear growth model showed an acceptable fit to the data, ꭓ^2^ (10) = 21.73, *p* = .017; RMSEA = .069; CFI/TLI = .960. Next, predictors were added to the linear growth model, resulting in a model with an acceptable model fit, ꭓ^2^(31) = 59.80, *p* = .001; RMSEA = .061; CFI = .942; TLI = .916. The results are shown in Supplementary Table 3. This model accounted for 44% of the variance in Mental Fatigue in the acute phase, and 11% of the variance of the decline in fatigue over time. With regard to the intercept, burn survivors with higher levels of acute PTSD symptoms reported higher levels of fatigue. With regard to the slope, burn survivors who reported extreme pain or discomfort in the acute phase showed a significantly less steep decline in Mental Fatigue over time than those with no or moderate pain or discomfort.

To investigate the relevance of the predictors for long-term symptoms of Mental Fatigue, we reran the model to predict the endpoint at 18 months post-burn instead of the intercept (acute phase). This adjusted model accounted for 25% of the variance in Mental Fatigue at 18 months post-burn. Supplementary Table 3 showed that burn survivors with extreme levels of acute pain/discomfort and those with higher acute PTSD symptoms, reported higher levels of Mental Fatigue at 18 months post-burn compared to those who reported no or moderate acute pain or lower acute PTSD symptoms respectively.

**Notes**

- The MFI-20 operationalized ‘Mental Fatigue’ as concentration problems.
- Note that in the main article, the General Fatigue score differs significantly from the population norms at 12 months post-burn. This result differs from what is reported here, due the different number of t-tests that is performed (25 vs 5), and hence a different correction factor for the p-values, leading to a different conclusion.

**References Supplemental Material**

Raykov, T., Marcoulides, G. A., & Millsap, R. E. (2013). Factorial Invariance in Multiple Populations:A Multiple Testing Procedure. *Educational and Psychological Measurement, 73*(4), 713-727. doi:10.1177/0013164412451978

Schwarz, R., Krauss, O., & Hinz, A. (2003). Fatigue in the general population. *Onkologie, 26*(2), 140-144. doi:10.1159/000069834

Smets, E. M., Garssen, B., Bonke, B., & De Haes, J. C. (1995). The Multidimensional Fatigue Inventory (MFI) psychometric qualities of an instrument to assess fatigue. *J Psychosom Res, 39*(3), 315-325. doi:10.1016/0022-3999(94)00125-o
