# Supplementary figures and images for: Bio-Psychological Predictors of Acute and Protracted Fatigue After Burns: A Longitudinal Study
Source: Front Psychol. 2022 Jan 24;12:794364. doi: 10.3389/fpsyg.2021.794364 (PMC8818679; doi:10.3389/fpsyg.2021.794364)

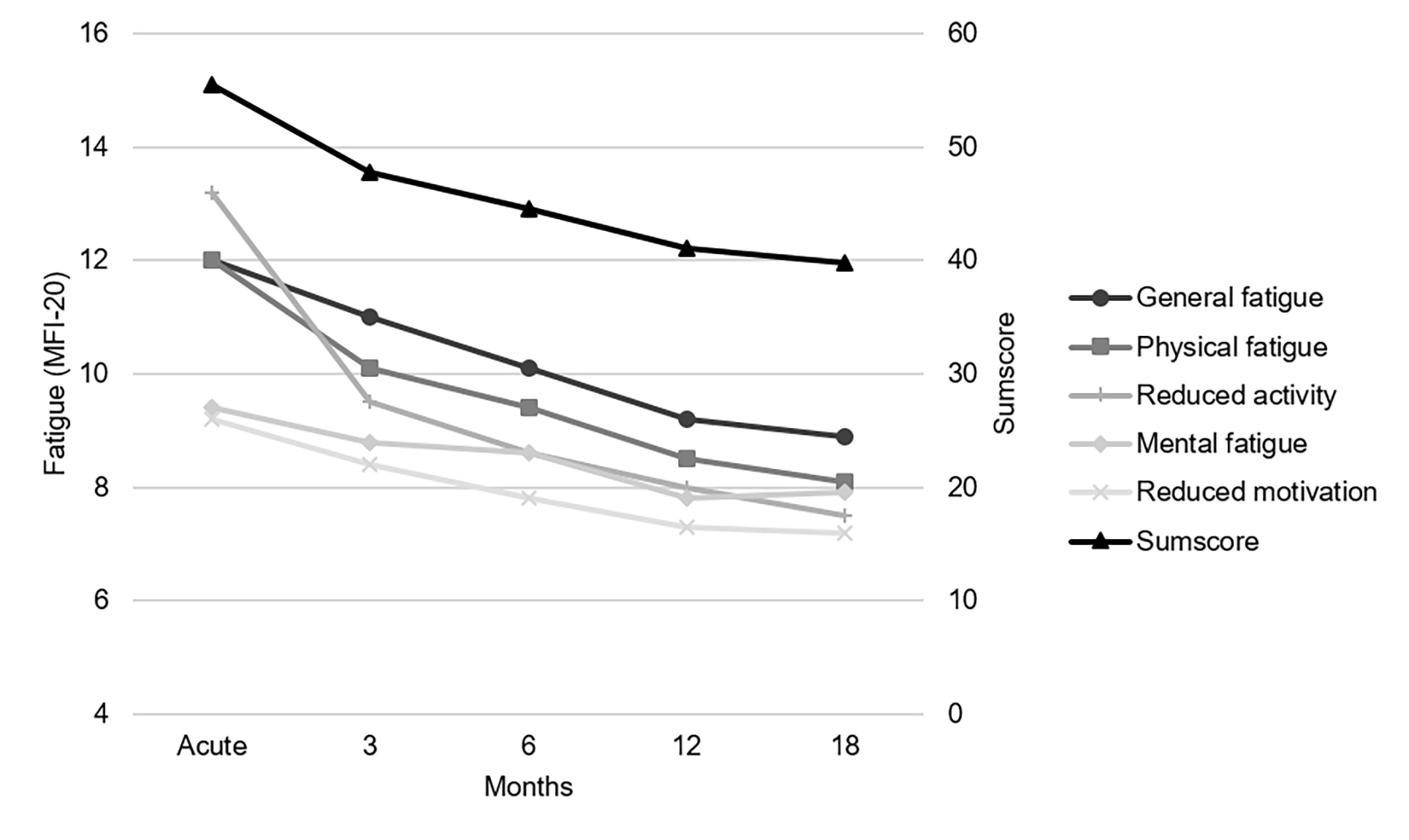

Supplement: Supplementary file 2 [file Image_1.tif]
